# Reproductive Immunology and Pregnancy 2.0

**DOI:** 10.3390/ijms25105132

**Published:** 2024-05-09

**Authors:** Dariusz Szukiewicz

**Affiliations:** Department of Biophysics, Physiology & Pathophysiology, Faculty of Health Sciences, Medical University of Warsaw, 02-004 Warsaw, Poland; dszukiewicz@wum.edu.pl or dariusz.szukiewicz@wum.edu.pl

This Special Issue comprises original articles in the field of clinical studies whose major topics concern the genetic and immunological aspects of miscarriage and pre-eclampsia, the isolation of decidua macrophages and Hofbauer cells in the placenta for diagnostic purposes, and epigenetic mechanisms that trigger labor. Similar topics will be covered in the third edition of this series of Special Issues (“Reproductive Immunology and Pregnancy 3.0”) [1].

The appropriate level of activity for many genes in the uterine spiral arteries, as well as decidual changes in the endometrium and placenta determine the optimal development of vessels at the site of implantation and fetal growth and development [2]. Genes responsible for proper vascularization at the implantation site are mainly related to the synthesis of representatives of two families of growth factors, i.e., the vascular endothelial growth factor (VEGF) family and the angiopoietin/tyrosine kinase receptor (TEK) family [3]. Low levels of *VEGF* and *ANGPT2* transcripts were reported in cases of pregnancies complicated by fetal growth restriction (FGR). Chronic placental hypoxia, the features of which were histopathologically confirmed, was accompanied by increased placental growth factor (PGF) expression, which may indicate a compensatory mechanism that was ineffective in this patient [4].

An increasing number of studies indicate that human endogenous retrovirus (HERV) genes play a significant role in placental pathology. *HERVs* constitute up to 8% of human genomic DNA and have been termed “fossil viruses” because they represent footprints of previous retroviral infections [5]. The placenta actively expresses a number of HERV genes [6]. For example, HERV-W env (syncytin-1), a captive retroviral envelope protein, is a membrane glycoprotein that is crucial for placental morphogenesis. Syncytin-1 is involved in fusogenic processes, during which cytotrophoblast cells continuously fuse with the overlying syncytiotrophoblast layers to establish barrier functions and transport activities [7]. In addition, the nonfusogenic effects of syncytin may be responsible for pathological placental morphogenesis based on increased apoptosis and proliferation [8]. Disturbed syncytin-1 expression is associated with increased risk of infertility, pre-eclampsia and FGR, but it has also been observed in patients with tumors such as neuroblastomas, endometrial cancer, and endometriosis [8,9]. Poor placental angiogenesis is a typical histopathological feature of syncytin-1 functional disruption and is often found in patients with pre-eclampsia [10]. Therefore, there are many arguments that syncytin-1 may be a new biological marker and a potential therapeutic target.

Clinically significant abnormal vascularization of placental tissue may be caused by reduced expression/deletion of the basic helix–loop–helix ARNT-like 1 (BMAL1) gene [11]. As the core circadian locomotor output cycle kaput (CLOCK) gene, *BMAL1* is rhythmically expressed in many tissues, including uterine and placental tissues, and is responsible for controlling the circadian expression of numerous target genes involved in many physiological processes [11,12]. It has been suggested that reduced endometrial *BMAL1* expression is associated with an increased risk of spontaneous recurrent miscarriages in humans [13,14]. Decidual cells deficient in *BMAL1* expression showed inhibitory effects on trophoblast invasion into uterine spiral arteries [15]. These findings at the molecular level should prompt a reassessment of the systemic consequences of disruption of the biological clock, especially with regard to fertility [16]. Moreover, the insufficient invasion of trophoblast cells, which is the main factor involved in the development of pre-eclampsia, is caused by the impaired activity of natural killer (NK) cells [14,15]. Therefore, if the cytolytic activity in NK cells required for trophoblast invasion follows a daily rhythm, it may be disrupted as a result of decreased *BMAL1* expression [17].

Because NK cells are the most abundant immune cells in the uterus, some researchers suggest that that the immune communication between the fetus and mother is moderated primarily by natural killer (NK) cells rather than T cells [18]. Notably, based on cytokine production, the T helper type 1 (Th1)/Th2 balance in PE gradually shifts to an NK1/NK2 balance [18,19]. The central role of NK cells in PE may be due to the fact that decidual NK (dNK) cells have a cytokine profile that is favored by the presence of HLA-E and HLA-C, and contributes to vascular remodeling and trophoblast invasion into the uterine spiral artery with the subsequent development of the placenta, whereas the cytotoxic phenotypes of peripheral or circulating NK (cNK) cells may be crucial for successful immune escape from fetal to maternal immunity at the maternal–fetal interface [20]. Vascular remodeling in PE is accompanied by an increased number of both dNK and cNK cells, exhibiting increased cytolytic activity, which is measured according to the intracellular production of interferon γ (IFN-γ), perforin, and granzyme B [21]. The increased number of NK cells may also be a manifestation of a compensatory mechanism in the event of the insufficient activation of dNK cells, as is the case in an improper combination of killer cell immunoglobulin-like receptors (KIRs) and human leukocyte antigens (HLA)-C expressed by extravillous trophoblast cells (EVTs) [19,22]. The involvement of NK cells in the pathomechanism of PE is shown in Figure 1.

The results from the latest research on the molecular mechanisms of miscarriage have sparked interest in regulatory T cells (Tregs) as central modulators of the intensity of the immune response in the maternal–fetal system. This specific role of Tregs is to maintain homeostasis during pregnancy by suppressing the activation of autoreactive or alloreactive effector T cells through a diverse repertoire of molecular mechanisms [24,25]. The application of Treg epitopes capable of inducing tolerance seems to be a promising approach for the treatment of immunological causes of pregnancy loss [26,27]. Moreover, the lack of immunogenicity of Treg epitopes in vivo means that they should prove safe for future clinical applications in humans [28,29]. However, the selection/creation of such epitopes is challenging because predicting the action of a given epitope is difficult due to the high degree of MHC polymorphism and disparity in the volume of data on various steps encountered in the generation and presentation of T-cell epitopes in living systems [30].

Among the immune system cells in the human placenta, the dominant type in terms of number and importance are macrophages. The macrophages on the maternal side (decidual) and the macrophages on the fetal side (Hofbauer cells) of the placenta show phenotypic differences associated with changes in activity (polarization). The precise determination of the polarization of placental macrophages in physiological and complicated pregnancies may provide valuable information, as the important role of these immune cells in implantation, placentation and throughout the course of pregnancy is unquestionable [31,32]. The development of a representative isolation method for the direct comparison of maternal and fetal macrophages may shed new light on both the physiology and immunopathology of the placenta, possibly leading to the development of new therapeutic approaches [33].

## Figures and Tables

**Figure 1:**
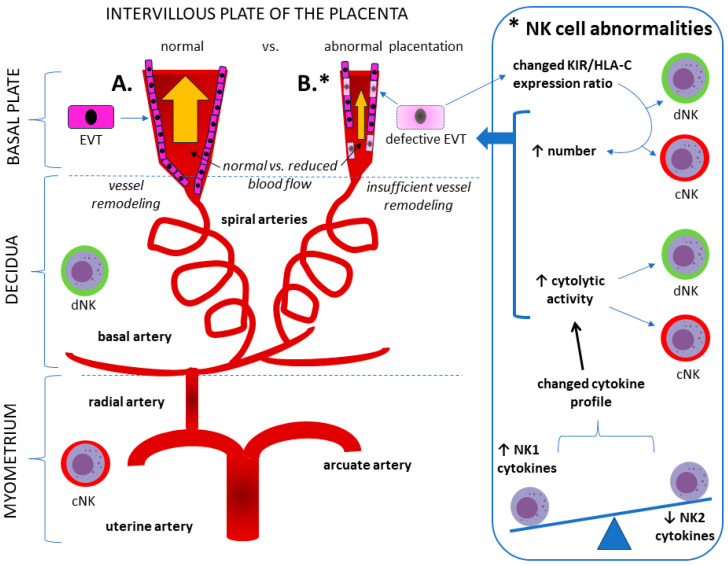
The concept of the pathomechanism of pre-eclampsia (PE) based on the central role of natural killer (NK) cells. (**A**). **NORMAL PREGNANCY.** A sufficiently deep invasion of extravascular trophoblast (EVT) cells into the uterine spiral arteries determines proper implantation, creating low-resistance circulation with optimal blood flow for the progression of pregnancy growth. Bold is used intentionally for better readability and clear differentiation between parts A and B of Figure 1. (**B**). **PRE-ECLAMPSIA.** A significant immune system disorder in PE patients is a change in the number and activity of NK cells. This phenomenon applies to both decidual NK (dNK) cells and peripheral (circulating; cNK) cells. The increase (↑) in the number of NK cells may be a manifestation of a compensatory mechanism in the event of the insufficient activation of NK cells, which may be caused by a change in the proportion of killer cell immunoglobulin-like receptor (KIR) expression on NK cells relative to human leukocyte antigen c (HLA-C) in defective extravillous trophoblast (EVT) cells [23]. The change in NK cell activity may result from an imbalance between NK type-1 (NK1) and NK2 cells, as pregnant women with PE have a significantly greater NK1/NK2 cell ratio than healthy pregnant women. This leads to the dominance of NK1, which is manifested by a change in the cytokine profile toward increased cytotoxic and cytolytic activity. The presence of defective EVT cells causes abnormal placentation with insufficient vessel remodeling, preventing the creation of low-resistance circulation between the spiral uterine arteries and the intervillous placental plate vessels.

## Data Availability

No new data were created or analyzed in this study. Data sharing is not applicable to this article.
